# Chimeric antigen receptors (CARs) incorporating mutations in the IgG4 Fc spacer region to eliminate Fc receptor recognition results in improved CAR T cell persistence and anti-tumor efficacy

**DOI:** 10.1186/2051-1426-1-S1-P18

**Published:** 2013-11-07

**Authors:** Mahesh Jonnalagadda, Armen Mardiros, Lauren Hoffman, Alyssa Bernanke, Wen-Chung Chang, William Bretzlaff, Renate Starr, Xiuli Wang, Julie Ostberg, Christine Brown, Stephen J Forman

**Affiliations:** 1 Hematology & Hematopoietic Cell Transplantation, Beckman Research Institute and City of Hope National Medical Center, Duarte, CA, USA

## 

Adoptive immunotherapy using T cells genetically redirected via expression of chimeric antigen receptors (CARs) is a promising approach for cancer treatment. However, this immunotherapy is dependent in part on the optimal molecular design of the CAR, which involves an extracellular ligand-binding domain connected to an intracellular signaling domain by spacer and/or transmembrane sequences. CAR designs frequently incorporate extracellular linker regions based on the immunoglobulin constant regions of either IgG1 or IgG4. In this study we evaluated the potential for the IgG4-Fc linker to result in off-target interactions between the CAR and Fc gamma receptors (FcγRs). As proof of principle, we have focused on a CD19-specific CD19scFv-IgG4-CD28-zeta CAR, and indeed found that CAR+ T cells bound to soluble FcγRs, and did not engraft in NSG mice compared to CAR-negative T cells that only expressed an EGFRt tracking marker. We hypothesized that mutations to avoid FcγR interactions would improve CAR+ T cell persistence and anti-tumor efficacy. To this end, we generated a CD19-specific CAR that has been mutated at two sites within the CH2 region (L235E; N297Q) of the IgG4 Fc spacer, here called CD19R(EQ), as well as a CD19-specific CAR that has a CH2 deletion in its IgG4 Fc spacer (CD19Rch2Δ). These mutations/deletion do not alter the functional ability of the CAR, when expressed by T cells, to mediate antigen-specific lysis of tumor cells. However, compared to T cells that express a non-mutated CAR, T cells expressing the CD19R(EQ) and CD19Rch2Δ exhibit impaired binding to recombinant soluble FcγRs. These CD19R(EQ) and CD19Rch2Δ T cells also exhibit improved engraftment in NSG mice. Indeed the engraftment levels seen with the mutated CAR were similar to that seen with CAR-negative T cells that only expressed the EGFRt tracking marker. Importantly, elimination of CAR/FcγR interactions also significantly improves CD19-specific CAR+ T cell anti-lymphoma efficacy in NSG mice. These studies provide evidence that optimal CAR function necessitates the elimination of cellular FcγR interactions in order to improve T cell persistence and anti-tumor responses.

